# A supratentorial primitive neuroectodermal tumor presenting with intracranial hemorrhage in a 42-year-old man: a case report and review of the literature

**DOI:** 10.1186/1752-1947-7-86

**Published:** 2013-03-27

**Authors:** Evangelos K Papadopoulos, Kostas N Fountas, Alexandros G Brotis, Konstantinos N Paterakis

**Affiliations:** 1 Department of Neurosurgery, University Hospital of Larissa, School of Medicine, University of Thessaly, Building A, 3rd Floor, Biopolis, Larisa, 41110, Greece

## Abstract

**Introduction:**

We report on a very rare case of a supratentorial primitive neuroectodermal tumor in an adult, which presented with intracerebral hemorrhage, and review the relevant medical literature.

**Case presentation:**

A 42-year-old Caucasian man complained of a sudden headache and nausea-vomiting. The patient rapidly deteriorated to coma. An emergency computed tomography scan showed an extensive intraparenchymal hemorrhage that caused significant mass effect and tonsilar herniation. During surgery, an increased intracranial pressure was recorded and extensive bilateral decompressive craniectomies were performed. A cherry-like intraparenchymal lesion was found in his right frontal lobe and resected. The patient died in the intensive care unit after approximately 48 hours. The resected lesion was identified as a central nervous system primitive neuroectodermal tumor.

**Conclusion:**

Supratentorial primitive neuroectodermal tumors must be considered in the differential diagnosis of space-occupying lesions in adults. Spontaneous supratentorial hemorrhage due to primitive neuroectodermal tumors is an extremely rare but potentially lethal event.

## Introduction

Primitive neuroectodermal tumors (PNET) constitute a heterogeneous group of embryonal malignancies that are composed of undifferentiated or poorly differentiated cells and are characterized by an aggressive clinical behavior [1,2]. They usually occur in children and adolescents and may arise in the cerebral hemispheres, the brainstem or the spinal cord [1,3]. Although they are common primary central nervous system tumors in children, PNETs are extremely rare in adults, representing less than 0.5% of all intracranial tumors [4]. The definition of PNETs in the 2007 World Health Organization (WHO) classification of tumors affecting the central nervous system is made regardless of their supra- or infratentorial location [2].

PNETs in adults usually present with signs of increased intracranial pressure (ICP), seizures and/or focal neurological deficits [4-15]. However, to the best of our knowledge, there is no report on a supratentorial PNET presenting with a massive and fatal intracerebral hemorrhage. In this case report, we present the case of a patient with a supratentorial PNET, presenting with massive intracerebral hemorrhage. With this opportunity, we also review the pertinent literature.

## Case presentation

**Figure 1:**
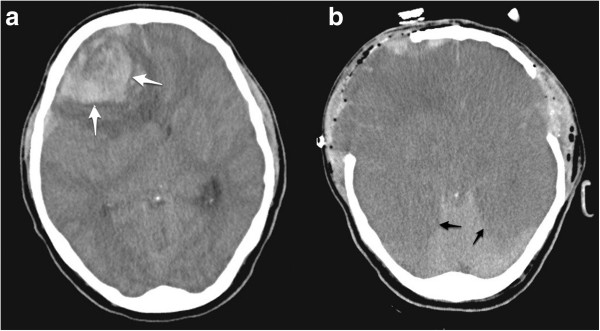
**Computed tomography scan images.** (**a**) Preoperative brain computed tomography scan showing an extensive intraparenchymal hemorrhagic lesion in the right frontal lobe (white arrows), with severe diffuse perilesional edema affecting the whole ipsilateral hemisphere, and compressing the ipsilateral lateral ventricle and causing significant mass effect. (**b**) Immediate postoperative computed tomography scan showing the low-density appearance of both the cerebral hemispheres, suggesting severe brain ischemia (black arrows).

A 42-year-old Caucasian man was admitted to the emergency department of a district hospital in a comatose condition (Glasgow Coma Scale Score 4 out of 15). His relatives reported that he initially complained of a sudden onset of headache and had several episodes of emesis. He was sedated, intubated and urgently transported to our teaching hospital. On arrival at our facility, he was unresponsive to verbal and painful stimuli, and his pupils were bilaterally dilated and non-reactive. An urgent computed tomography (CT) scan of his brain showed an extensive intraparenchymal hemorrhagic lesion, causing significant mass effect and tonsilar herniation (Figure 1a).

**Figure 2:**
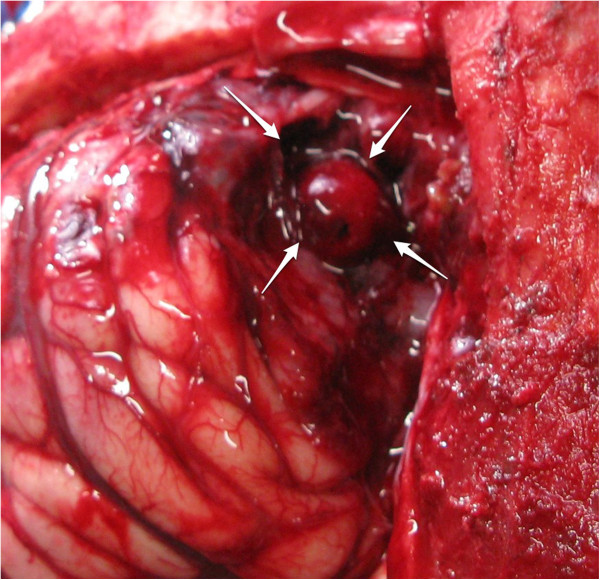
**Intraoperative photograph.** A cherry-like round lobulated lesion was demonstrated in the right frontal lobe (white arrows).

An external ventricular drain was inserted into his left lateral ventricle, and an intraparenchymal ICP fiber optic monitor was inserted into the right frontal perilesional area. His ICP was elevated (>40mmHg), and extensive bilateral decompressive craniectomies were urgently performed. During the surgery, and after the evacuation of the hematoma, a cherry-like intraparenchymal lesion was found in his edematous right frontal lobe, which was resected (Figure 2). Decompressive duraplasties were performed bilaterally and after surgery he was transferred to the intensive care unit for observation and further management.

A head CT scan at the end of surgery showed diffuse areas of low-density in both brain hemispheres, suggesting severe ischemia (Figure 1b). His condition continued to deteriorate, with ICP rising to 65mmHg despite the performed decompressive craniectomies and the aggressive medical management of his intracranial hypertension. He subsequently developed multi-organ failure, and died approximately 48 hours after his admission.

**Figure 3:**
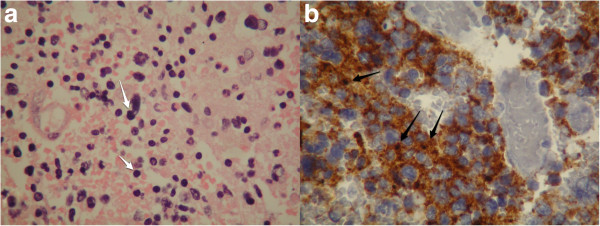
**The histological findings were compatible with primitive neuroectodermal tumor.** (**a**) Photomicrograph (original magnification, ×100; hematoxylin-eosin stain) demonstrating small tumor cells with round to oval hyperchromatic nuclei and scant cytoplasm with distinct outline (white arrows). (**b**) Photomicrograph (original magnification, ×400; immunohistochemical stain) showing intense positivity for synaptophysin (black arrows).

The surgical specimen was sent for microscopic and immunohistochemical pathological analysis. Permanent specimen sections were stained with hematoxylin and eosin and were assessed via immunohistochemistry for vimentin, synaptophysin, glial-fibrillary acidic protein and Ki-67 to measure the proliferation index. Uniform neoplastic medium-sized cells, with round to oval hyperchromatic nuclei, were present and positive for vimentin, synaptophysin and cluster of differentiation 56, but were negative for glial-fibrillary acidic protein (Figure 3). The Ki-67 index was approximately 40%, indicating increased mitotic activity and a high-grade malignancy. The resected lesion was identified as a central nervous system PNET.

## Discussion

The term PNET was introduced in 1973 for describing initially supratentorial, pediatric brain tumors that were largely undifferentiated or primitive but contained small foci of glial or neuroblastic differentiated cells of primitive neuroectodermal origin [1]. PNETs of the central nervous system include neuroblastomas, ganglioneuroblastomas, medulloepitheliomas, ependymoblastomas, embryonal tumors with abundant neuropil and true rosettes, and not otherwise specified PNET tumors [2]. The previous long-standing controversy regarding the inclusion of medulloblastomas and pineoblastomas into the PNET category has been resolved by the WHO tumor classification [2].

We performed a literature review in MEDLINE and PubMed, limited to the English language and ranging from January 1973 to December 2012. We used as key terms the words ‘PNET’ or ‘primitive neuroectodermal tumour’, intracerebral hemorrhage’ or ‘intracerebral haemorrhage’ or ‘intracerebral bleeding’ and ‘adults’, ‘man’, ‘woman’. No patients younger than 20 years old were included in the literature review. Altogether, 278 articles were retrieved. Two of the authors (EKP and AGB) read the abstracts of the gathered articles and all irrelevant articles were eliminated. The full-text articles of the remaining hits were gathered and thoroughly studied and their references scrutinized for further relevant cases. The results are presented in Table 1.

**Table 1:** Summary table of case reports on adult patients with supratentorial primitive neuroectodermal tumor

**Study**	**Number of patients**	**Age (years)**	**Sex**	**Clinical presentation**	**Intracerebral hemorrhage**	**Treatment**	**Outcome**
Bellis *et al*. [5]	1	57	M	Seizures	No	S, R	Died after five months
Gaffney *et al*. [6]	7	22 to 35	2F+5M	Increased ICP	No	S, ±R, ±C	Seven months to nine years
Kuratsu *et al*. [7]	1	31	M	Headache, hemiparesis, papilledema	No	S, C	Died after 15 months
Louis *et al*. [16]	1	40	M	Hydrocephalus	No	S, R	Spinal metastasis after 18 years
Miyazawa *et al*. [17]	1	21	F	Headache	No	S, R, C	27 years follow-up
Selassie *et al*. [18]	1	60	M	Visual difficulty	No	S, R, C	Died after eight months
Pickuth and Leutloff [8]	5	20 to 57	2F+3M	Increased ICP	No	S, ±R, ±C	Died after four to 25 months
Sanno *et al*. [9]	1	50	F	Seizures	No	S	NS
Takeuchi *et al*. [19]	1	69	M	Decline of higher cortical functions	No	S, R, C	30 months follow-up
Gyure *et al*. [20]	4	30 to 40	4M	Various	No	S, ±R, ±C	Died after two to five years
Prados *et al*. [21]	4	21 to 42	3M+1F	Not specified	No	S, R, C	23 to 66 months
Gnanaligham *et al*. [22]	1	38	F	Vertigo - diplopia	No	S	Died after five months
Krishnamurthy *et al*. [23]	1	51	F	Visual field defects	No	S, R, C	Alive at 18 months follow-up
Balafouta *et al*. [10]	1	60	M	Headache, Broca’s aphasia and hemiparesis	No	S, R, C	12 months
Majós *et al.*[24]	7	21 to 67	NS	NS	No	NS	NS
Kim *et al*. [4]	12	20 to 64	6M+6F	Increased ICP	No	S, ±R, ±C	Mean survival 86 months
Kouyialis *et al*. [25]	1	32	F	Headaches, fatigue, gait disturbances	No	S	Alive at 24 months
Shingu *et al*. [11]	1	88	F	Disturbance of consciousness, left hemiplegia	No	Biopsy, C	Died after six months
Krampulz *et al*. [12]	1	33	M	Seizures, dizziness	No	S, R, C	Alive after 17 years
Han *et al*. [13]	1	29	M	Increased ICP	No	S, R, C	Died after two years from spine and lung metastases
Terheggen *et al*. [26]	1	26	M	Headache, nausea, coordination problems and monoparesis	No	S, R	Multiple liver metastases and local tumor recurrence
							Alive after four years
Ohba *et al*. [14]	1	56	M	Facial palsy	No	S, R	Died after five months
Prayson [27]	1	48	F	Headaches and confusion	No	S	Recurrence after 10 months
							Died after 13 months
Gessi *et al*. [28]	12	32 to 64	6F+6M	NS	No	NS	Varied from death at two months to five years disease free
Lawandy *et al*. [15]	1	22	M	Increased ICP	No	S	NS
Present case	1	42	M	Coma	Massive intracerebral hemorrhage	S	Death

+/−: on some cases; *C* chemotherapy, *F* female, *ICP* intracranial pressure, *M* male, *NS* not specified, *R* radiotherapy; *S* surgery.

Twenty six publications have reported on 70 supratentorial not otherwise specified PNET cases in adults. These tumors occurred in adults up to 88 years old [11] with a small overall male predominance (male to female ratio, four to three) (Table 1).

Most patients present with symptoms and signs of raised ICP such as headaches, nausea and/or vomiting, confusion and papilledema because of the rapid tumor growth [4,6-8,13,15], as in our case. Furthermore, an adult supratentorial PNET may present with focal neurological signs and deficits, such as limb paresis, aphasia, facial palsy and visual field defects, depending on the anatomic location of the tumor [7,10,11,14,18,22,23,26]. Occasionally, the clinical presentation may involve seizures [5,9,12]. To the best of our knowledge, this is the first report of a patient with a supratentorial PNET presenting in a comatose condition due to massive intracranial hemorrhage.

Adult PNETs demonstrate no specific imaging characteristics in comparison to their counterparts in children. They may appear as isodense or hyperdense lesions on a CT scan, with intratumoral calcifications or microhemorrhages [4]. On magnetic resonance imaging, they usually appear as multilobulated, well-demarcated masses, showing heterogeneous signal intensity on T1 and T2 weighted images. They may have cystic, necrotic or hemorrhagic components, while their solid parts usually appear hypointense on T1-weighted and hyperintense on T2-weighted images, and they enhance after intravenous administration of a paramagnetic contrast. Moderate to severe surrounding edema is usually present in adult supratentorial PNETs [4]. Proton magnetic resonance spectroscopic studies have shown nonspecific increased choline and lactate, and decreased N-acetyl-aspartate concentrations. The differential preoperative diagnosis of suspected supratentorial PNET should include glioblastoma, anaplastic oligodendroglioma and metastatic tumor. It has to be emphasized that the possibility of mislabeling a diffuse highly malignant glioma with foci resembling PNET is extremely high [3].

On macroscopic examination, adult PNETs usually appear as lobulated, purple-grayish or pinkish masses. On histology, the tumor cell population consists of undifferentiated small cells with ill-defined, scanty cytoplasm, and round or oval cells with hyperchromatic nuclei [2,3]. Mitotic activity is variable. Microscopic calcifications, necroses and Homer-Wright rosettes are also observed in a number of cases. The vascularity of the tumors varies, whereas endothelial cell proliferation within the vessel wall is regularly observed [2,3,15].

Newer genetic analysis studies have demonstrated that PNETs are characterized by *MYCN* or *MYCC* gene amplifications, and polysomies of chromosomes 2 and 8 [3]. Various genetic features seem to be associated with prognosis and clinical outcome. The presence of *MYCN* or *MYCC* gene amplifications has been shown to be associated with decreased survival. Similarly, polysomies of chromosomes 2 and 8 have been individually associated with decreased survival in children, and the combined presence appears to be an even more unfavorable prognostic factor [3]. The vast majority of the genetic characteristics are based on pediatric cases because adult cases are extremely rare. Therefore, extrapolation of the prognostic role of such genetic features in PNET cases in adults may be inaccurate.

Extensive surgical resection of adult PNET is the usually recommended mode of treatment, followed by radiotherapy and chemotherapy (Table 1). Postsurgical irradiation of the whole craniospinal axis may be employed, even when there is no evidence of spinal seeding [4]. The employed mean dose is 54Gy for the brain and 31Gy for the spinal cord [4]. The exact role of stereotactic radiosurgery employment, especially in cases of partially resected PNET tumors, remains to be defined [2,4].

The prognosis for adults with supratentorial PNETs seems to be worse than that of their pediatric counterparts [3-5,7,29]. The reported overall survival of PNETs in the pediatric population ranges between 29% and 57% [3], whereas Kim *et al*. [4] reported three-year mean survival of 75% in their series of adult patients. Most adults, with limited exceptions, with a supratentorial PNET die within a year of the initial diagnosis (Table 1). There are a few reports on local recurrence and spinal and lung metastases [13,16,26,27]. For pediatric patients with supratentorial PNETs, the prognosis correlates with age, extent of necrosis and tumor dissemination [4]. By contrast, in adults the most important prognostic factor is considered to be the Ki-67 index. Kim *et al*. [4] reported that adult patients with a Ki-67 index greater than 30% demonstrated very poor outcome, with a mean postoperative survival time of eight months. Similarly, in our patient, his labeling index Ki-67 was approximately 40%.

## Conclusions

Supratentorial PNETs must be considered in the differential diagnosis of space-occupying lesions in adults. Spontaneous supratentorial hemorrhage is an extremely rare but potentially lethal presentation of PNET in adults.

## Consent

Written informed consent was obtained from the patient’s relatives for publication of this case report and accompanying images. A copy of the written consent is available for review by the Editor-in-Chief of this journal.

## Competing interests

The authors declare that they have no competing interests.

## Authors’ contributions

All authors contributed in writing the manuscript. EKP and AGB performed the literature review. KNF was responsible for the text editing. AGB was responsible for writing the manuscript. KNP was in charge of manuscript supervision and is the corresponding author. All authors read and approved the final manuscript.
